# Development of a reliable and accurate algorithm to quantify the tumor immune stroma (QTiS) across tumor types

**DOI:** 10.18632/oncotarget.22932

**Published:** 2017-12-04

**Authors:** Rainer Christoph Miksch, Jingcheng Hao, Markus Bo Schoenberg, Katharina Dötzer, Friederike Schlüter, Maximilian Weniger, Shuai Yin, Steffen Ormanns, Jan Goesta D'Haese, Markus Otto Guba, Jens Werner, Barbara Mayer, Alexandr V. Bazhin

**Affiliations:** ^1^ Department of General, Visceral and Transplantation Surgery, Ludwig-Maximilians University, Munich, Germany; ^2^ Department of Pathology, Ludwig-Maximilians University, Munich, Germany; ^3^ German Cancer Consortium (DKTK), Partner Site Munich, Germany

**Keywords:** tumor-infiltrating lymphocytes, colorectal cancer, ovarian cancer, hepatocellular carcinoma, pancreatic cancer

## Abstract

The tumor microenvironment plays an important role in the tumor biology. Overall survival of tumor patients after resection is influenced by tumor-infiltrating lymphocytes (TILs) as a component of the tumor stroma. However, it is not clear how to assess TILs in the tumor stroma due to heterogeneous methods in different cancer types. Therefore, we present a novel Quantification of the Tumor immune Stroma (QTiS) Algorithm to reliably and accurately quantify cells in the tumor stroma. Immunohistochemical staining of CD3 and CD8 cells in sections of metastatic colorectal cancer (mCRC), ovarian cancer (OvCa), hepatocellular carcinoma (HCC), and pancreatic ductal adenocarcinoma (PDAC), alltogether *N* = 80, was performed. Hot spots of infiltrating immune cells are reported in the literature. Reliability of the hot spot identification of TILs was examined by two blinded observers. Accuracy was tested in 1 and 3 hot spots using computed counting methods (ZEN 2 software counting (ZC), ImageJ software with subjective threshold (ISC) and ImageJ with color deconvolution (IAC)) and compared to manual counting. All tumor types investigated showed an accumulation of TILs in the tumor stroma (peri- and intratumoral). Reliability between observers indicated a high level consistency. Accuracy for CD8+/CD3+ ratio and absolute cell count required 1 and 3 hot spots, respectively. ISC was found to be the best for paraffin sections, whereas IAC was ideal for frozen sections. ImageJ software is cost-effective and yielded the best results. In conclusion, an algorithm for quantification of tumoral stroma could be established. With this QTiS Algorithm counting of tumor stromal cells is reliable, accurate, and cost-effective.

## INTRODUCTION

In recent years the understanding of tumors regarding their dynamic proliferation, growth and so their composition has changed. It has become increasingly clear that malignant neoplasms are also influenced by particular cellular and non-cellular tumor components, so called tumor stroma. The tumor stroma influences carcinogenesis and tumor biology [1]. This is in part why a mere description of tumor burden, such as in the TNM tumor staging system, does not always have a high predictive and prognostic value [2, 3]. Therefore, immune cell infiltration, the most common examined tumor stromal cells have become a focus of intense research [4, 5]. In several tumor types it has been reported that stromal cells such as fibroblasts may also have a regulatory function in the biologic behavior of malignancies [6].

The immune components of the tumor stroma especially CD3^+^ and CD8^+^ infiltrating cells have been reported frequently in different tumor types [7, 8]. In fact, some studies suggest that peri- and intratumoral immune cell infiltration exceeds the established staging systems (i.e. TNM) in predictability [9]. Therefore, quantification of cancer infiltrating immune cells has been described as a new clinical score across different tumor types [1, 3, 10, 11]. Although many publications describe influence on survival, the methodological aspects such as sample preparation, description of sectioning, details of antibody staining, and counting methods have been often vague or not mentioned [9, 12, 13]. Contrary to that, there are quantification methods in immunohistochemistry (IHC) which are widely standardized. The Ki67 index for example is essential for neuroendocrine tumors in clinical practice. However, since its establishment different counting concepts were adopted. Because of these differences methodological studies were needed to identify the best counting methods [12–14]. Similarly, to provide predictive scoring of the tumor stroma across tumor types, the methods need to be well defined, reproducible and readily available. In this way results reported in the literature can be put into perspective and compared directly.

Based on this study a reliable and accurate algorithm to quantify the immune components of the tumor immune stroma across different tumor types (Hepatocellular carcinoma (HCC), pancreatic cancer (PDAC), ovarian cancer (OvCa), and metastatic colorectal cancer (mCRC)) is proposed.

## RESULTS

### Immunohistochemistry

**Figure 1:**
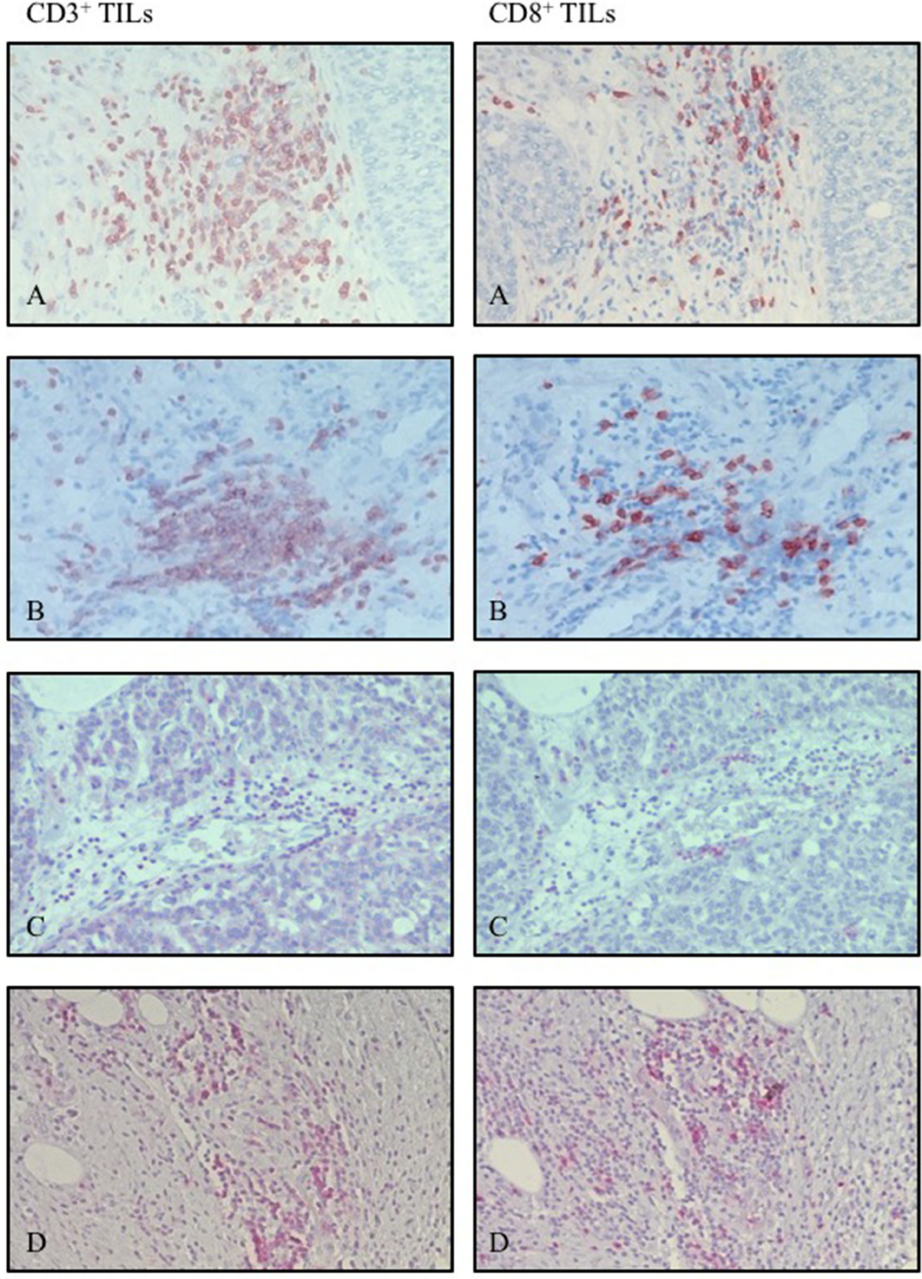
Representative hot spots of infiltrating CD3^+^ and CD8^+^ cells out of the same area using magnification of 20x (**A**) metastatic colorectal cancer, (**B**) ovarian cancer, (**C**) hepatocellular carcinoma, (**D**) pancreatic ductal adenocarcinoma).

**Figure 2:**
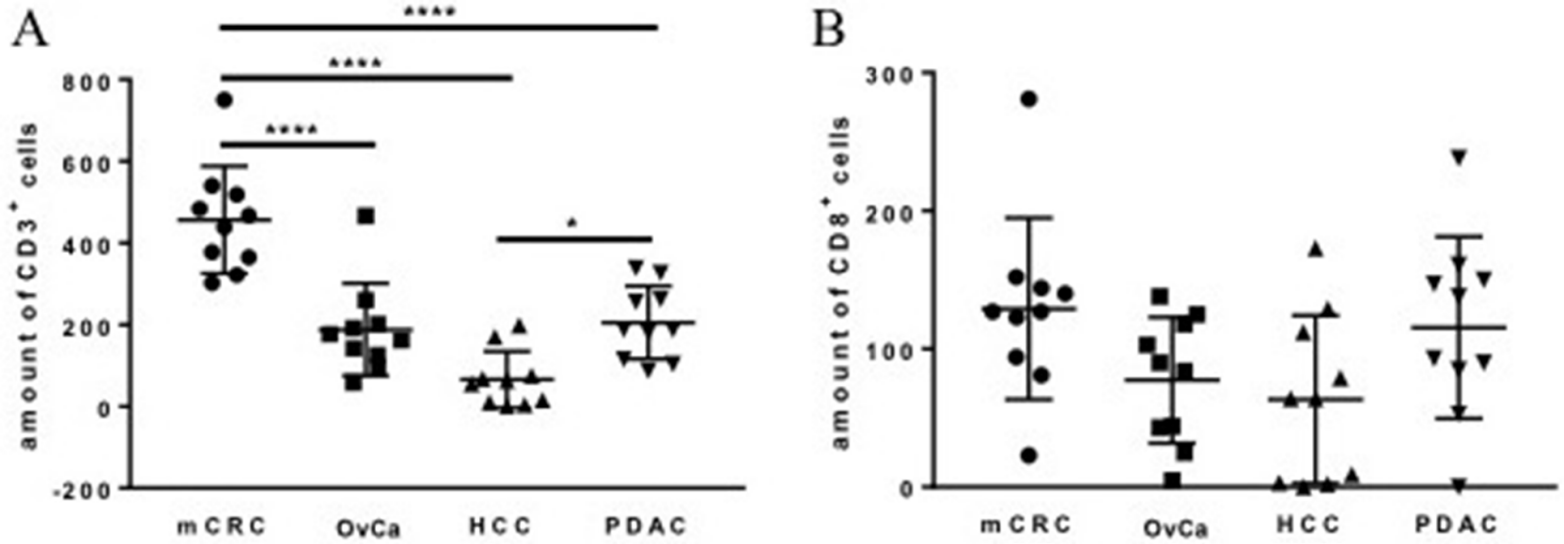
Amount of CD3^+^ (**A**) and CD8^+^ cells identified with IHC in tumor samples. The data of staining of 10 patients from each group are presented with SD and analyzed with the ordinary one-way ANOVA with Tukey’s multiple comparisons post test, ^*^p < 0.05 and ^****^ *p* < 0.0001: significant difference in the amount of T cells is shown.

Immunohistochemical analysis revealed a positive CD3 and CD8 staining in all cancer tissue sections of mCRC, OvCa, HCC, and PDAC (Figure 1). The cells were counted manually using 3 hot spots per slide and analyzed using descriptive statistics (Table 1). The highest level of CD3^+^ cell infiltration was found in mCRC samples, in PDAC and OvCa this level was intermediate and HCC samples showed the lowest level of the infiltration (Figure 2A). These differences were significant. No difference was found in the amount of the CD8^+^ cell infiltration in the tumor samples tested (Figure 2B). As expected amount of CD3^+^ cells were higher compared to CD8^+^ ones in mCRC, OvCa, and PDAC (Figure 2).

**Table 1:** Descriptive statistics of IHC analysis of tumor samples

Cell amount		mCRC	OvCa	HCC	PDAC
**CD3**	Minimum	302	59	0	88
	25% Percentile	354.3	118	8.5	112.3
	Median	453	169.5	59.5	189
	75% Percentile	524.3	216.5	98.75	279
**CD8**	Minimum	23	5	0	0
	25% Percentile	90.75	38.5	2.75	77
	Median	127	87	64	115.5
	75% Percentile	146	119.8	116.3	152.8

### Reliability analysis

Quantification results from 2 blinded observers for reliable detection of hot spots were compared using intraclass-correlation: 0.949 in mCRC, 0.843 in OvCa, 0.805 in HCC and 0.957 in PDAC. There was no significant difference in finding the largest hot spot in all tumor types comparing the 2 blinded observers (data not shown). Therefore, 1 observer showed high level of internal consistency.

### Accuracy analysis

**Figure 3:**
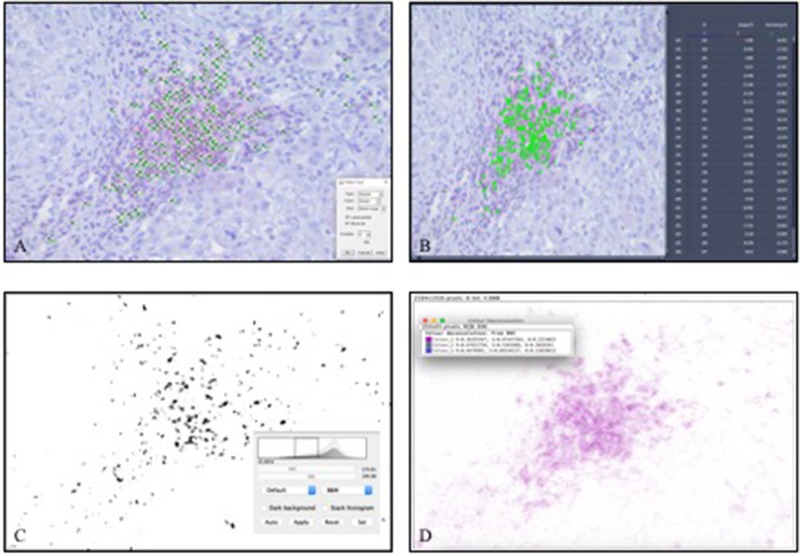
Manual counting and software-assisted counting methods shown representatively in one hot spot of infiltrating CD3^+^ T-lymphocytes in hepatocellular carcinoma (**A**) manual counting with ImageJ software, (**B**) Automated ZEN 2 software counting, (**C**) ImageJ software with subjective threshold, (**D**) ImageJ software with color deconvolution).

The CD8^+^/CD3^+^ ratio in 1 hot spot compared to the mean of 3 hot spots was consistent in all groups: ICC was 0.902 in mCRC, 0.908 in OvCa, 0.924 in HCC, and 0.885 in PDAC. The absolute cell count in 1 hot spot compared to the average in 3 hot spots did differ concerning regression coefficient B values over 1.2 for mCRC, OvCa, and PDAC (ICC scores: 0.973 in mCRC, 0.945 in OvCa, 0.963 in HCC, and 0.952 in PDAC). Comparison of the computed methods to the gold standard of manual counting showed mostly excellent accuracy (Figure 3 and Table 2). However, ZC in PDAC yielded inconsistencies with ICC = 0.601 and regression coefficient B = 1.280. ISC reached excellent results (> 0.900) in all groups (Table 2). IAC reached excellent accuracy in frozen sections of mCRC, OvCa, and HCC but not in PDAC (Table 2).

**Table 2:** Different methods of the staining analysis compared to manual counting

Methods		mCRC	OvCa	HCC	PDAC
**ZEISS - ZEN 2 blue: automated counting**	ICC	0.926	0.987	0.869	0.601
	B	0.868	0.968	0.621	1.28
**ImageJ: subjective threshold**	ICC	0.973	0.992	0.955	0.934
	B	0.851	1.03	0.723	0.914
**ImageJ: color deconvolution**	ICC	0.986	0.99	0.976	0.932
	B	0.945	1.06	0.791	1.327

### Counting time and costs

Furthermore, the counting time was compared for each tumor type and software (Table 3). Manual counting and ImageJ software with subjective threshold took most of the time, whereas time could be saved using computer assisted automatic counting methods (ZC and IAC). The presence of a microscope is required and presumed for each laboratory. The price for the hardware to connect the microscope and the computer was 2280.91€ (AxioVision, Carl Zeiss Inc., Germany). Whereas ImageJ can be downloaded for free, the ZEN 2 blue software costs 4152.64€. This amounts to 6433.55€ for the proprietary software-solution.

**Table 3:** Counting time in minutes for each software

	Manual counting		ZEISS - ZEN 2 blue		ImageJ: subjective threshold		ImageJ: color deconvolution	
	Median	Range	Median	Range	Median	Range	Median	Range
**mCRC**	10 min	1–12 min	1 min	1–2 min	10 min	5–14 min	6 min	4–7 min
**OvCa**	10 min	1–12 min	1 min	1–2 min	10 min	5–14 min	6 min	4–7 min
**HCC**	10 min	1–12 min	2 min	1–3 min	5 min	1–8 min	10 min	1–14 min
**PDAC**	8 min	1–12 min	1 min	1–2 min	4 min	1–9 min	2 min	1–3 min

## DISCUSSION

Evaluation of tumor tissue is mostly based on clinicopathologic staging systems. Nevertheless, tumor burden and further components of the tumor-microenvironment help to precise subtypes in different tumor types [9].

The tumor stroma which constitutes its microenvironment plays an important role in the understanding of tumor biology, progression, therapy, and lastly prognosis [1]. Especially, the immune system and its effector cells are known to influence prognosis [10]. For example higher numbers of CD3^+^ and CD8^+^ TILs have been shown to favorably influence prognosis of various tumor types [5, 7, 9, 15–21]. Different authors described classifications of infiltrating immune cells in breast cancer [22], lung cancer [23], and colorectal cancer [9]. Furthermore, they suggested that these classifications could amend the classical TNM system [9, 11, 22, 23].

In order for scoring systems to be clinically useful, standardization is key. However, in the literature counting methods and definitions are not clear and therefore results differ [9, 23, 24]. It complicates comparison of studies [9, 15]. For other quantification methods standards do exist and help guide clinicians during daily routine [12–14, 25–27].

**Figure 4:**
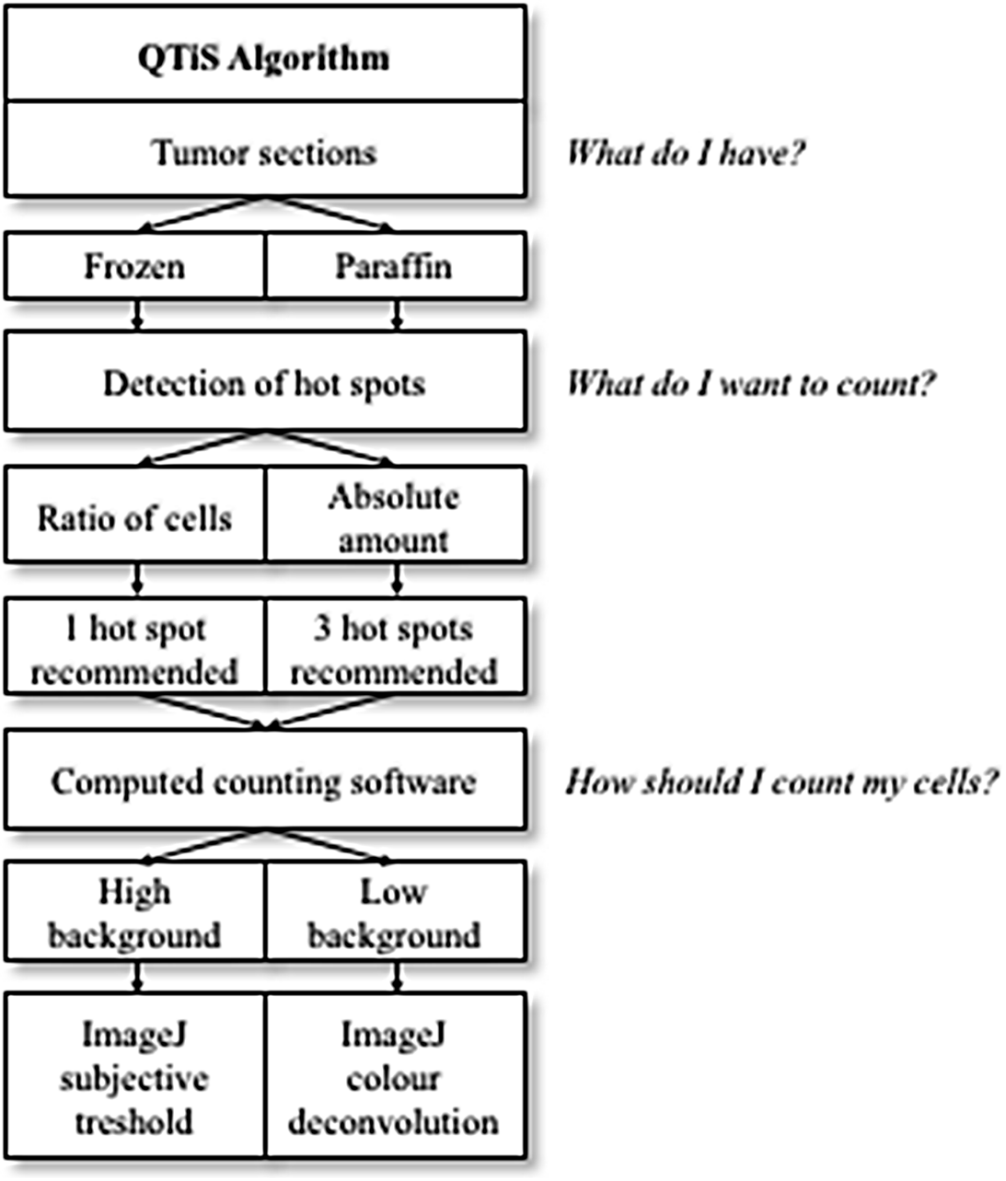
Quantification of the Tumor immune Stroma (QTiS) Algorithm: From the type of tumor sections to the final quantification of the tumor immune stroma

The aim of this study was to find the most reliable, accurate and affordable quantification of tumor immune stroma (QTiS) for routine clinical practice (Figure 4). In this study, the immune cell infiltration with CD3^+^ and CD8^+^ cells was used as the most widely examined representative of the tumor immune stroma [5, 7, 15–17, 28]. For other stromal cells, no such algorithm exists [29, 30].

The area with the highest density of CD3^+^ or CD8^+^ T-lymphocytes was defined as hot spot [31, 32]. Manual counting was regarded as the gold standard which was compared to computated software results. Actually, this underlying assumption is supported by the literature [12, 13]. Hot spot selection under the microscope was shown to be quicker at a lower cost than whole slide image scanning as reported in the literature [33, 34]. Furthermore, high demands of data processing and storage are needed when the whole slide is scanned [33, 35]. A typical scanned slide requires approx. 4.6 GB of memory [34], whereas a picture of 1 hot spot requires between 2.4 and 3 MB.

According to the subjective selection of hot spots, 1 observer is justified and reliable. This is also used in clinical practice – e.g. counting of Ki67 [12, 13]. If a ratio is to be determined - e.g. CD8^+^/CD3^+^, quantification of 1 hot spot compared to the mean of ratios of 3 hot spots was equitable. Ratios are often used to describe cell groups and subgroups [36]. It gives a quick impression of the consisting cells.

If the absolute cell count is to be determined, quantification of the mean of 3 hot spots is recommended and reliable. To give an absolute cell count over an area many researchers do not reveal their methods. It is not clear, if the counted area is representative for the tumor section [6, 9, 22, 23]. When using tissue microarray (TMA) at least the cores are punched out of the blocks [37] reflecting a smaller part of the tumoral tissue than in whole slides. The QTiS Algorithm (Figure 4) recommends counting of at least 3 hot spots per section. Others used up to 5 areas [39]. Actually, research about TILs is mostly focused on cell quantification in the tumor stroma with defined high/low infiltration groups and subgroups [5, 8, 9, 22, 28].

Currently, computed counting methods are able to achieve acceptable accuracy when compared to manual counting as gold standard [8, 12, 13]. ZEN and ImageJ software are by far not the only possible methods [6, 8, 9, 22–24, 31, 37]. As shown in our results background staining should be considered when selecting a method: ISC is accurate for sections with high background staining because it allows for human adjustments. IAC can be used in sections with low background staining. Furthermore, overlapping of cell layers may be a confounding factor. Therefore, subjective methods like ISC and IAC have an advantage over fully automated ZEN 2 software in helping differentiate cell clusters from single cells. Furthermore, ImageJ software is free and shows better results concerning cost and time – regarding training and experience - efficiency compared to ZEN 2 software and manual counting. Software used by other authors may differ and are dependent on access and funding [39].

This study has limitations: The algorithm to quantify the tumor immune stroma was performed with hot spots of CD3^+^ and CD8^+^ cells. On the one hand, these cell types present a small number of effector cells in the tumor stroma. On the other hand, CD3^+^ and CD8^+^ T-lymphocytes infiltrating tumor stroma are the most frequently published among different tumor types [5, 7, 15–18, 31, 37, 38, 40]. In this study 4 different counting methods were compared to each other statistically using ZEN and ImageJ software. There are far more counting methods described in the literature. Nevertheless, in this study examples of free and subjective software were compared to expensive and a fully automated counting software representatively. Lastly, this algorithm was developed in a limited set of samples. However, we used 80 different samples *n* = 10 for any marker and tumor type. With this sample size the QTiS Algorithm was statistically consistent. Furthermore, the results showed comprehensible and reproducible differences depending on the selected sections and high background staining in paraffin (HCC, PDAC) or frozen sections (mCRC, OvCa)).

With the newly developed QTiS Algorithm quantification of tumor immune stroma cells is reliable, accurate and cost effective.

## MATERIALS AND METHODS

### Materials

This study was approved and registered by the Human Tissue and Cell Research (HTCR) foundation (HCC: 2015-12, PDAC: 2016-04) and the Ethics Committee of the University of Munich (HCC: 395-16, PDAC: 807-16, OvCa: 278-04, mCRC: 252-04). For IHC staining of CD3 and CD8 frozen sections of mCRC and OvCa as well as paraffin sections of HCC and PDAC was used. Section preparation and selection were dependent on the current use of these tumor types in the laboratory: tissue of mCRC and OvCa has been available as frozen sections in our own laboratory, whereas tissue of HCC and PDAC has only been prepared as paraffin sections by the pathology department and HTCR. Staining was performed according to the type of section preparation. Altogether 80 sections of the 4 different tumor types were assessed. In each tumor type 20 slides were stained: 10 slides for CD3 and CD8 antigens each.

### Methods

#### Immunohistochemistry on frozen sections

Immunohistochemistry on frozen sections was described previously [41–43]. Briefly, after surgical removal, tumor samples were snap frozen in liquid nitrogen. Serial sections (5 μm) were prepared and fixed in acetone. Blocking of Fc receptors with 10% AB serum and endogenous biotin using the avidin-biotin blocking kit was performed. Primary antibodies against CD3 (clone UCHT1, mouse IgG1, working concentration: 1.25 μg/ml, source: Becton-Dickenson, NJ, US) and CD8 (clone C8/144B, mouse IgG1, working concentration: 3.0 μg/ml, source: Dako, Hamburg, Germany) were incubated for 60 min at room temperature. MOPC-21 (mouse IgG1, working concentration: 3 μg/ml, source: Sigma-Aldrich, Steinheim, Germany) was used as an isotype control. For detection, the Avidin-Biotin-complex staining method using a biotinylated antibody (rabbit anti mouse, polyclonal, dilution:1:2000, source: Dianova, Hamburg, Germany) and the horse-radish peroxidase labeled streptavidin (dilution:1:1000, source: Dianova) was performed according to the instruction of the manufacturer. 3-Amino-9-Ethylcarbozole was used as chromophore.

#### Immunohistochemistry on paraffin sections

Serial sections of 4 μm were used. Anti-CD3 antibody (ab5690, Abcam PLC, United Kingdom) and anti-CD8 antibody (ab4055, Abcam PLC, United Kingdom) were utilized briefly modified to IHC staining protocol after establishment with 1:50 antibody concentrations [44]. Antigen retrival for CD3 was performed with citrate buffer (pH = 6) for 30 minutes. CD8 with EDTA buffer (pH = 8) for 15 minutes was established. The temperature was 96°C for antigen retrival. Negative control was performed by replacing the antibody with 5% bovine serum albumin (BSA)/phosphate buffered saline (PBS). The antibody and negative control have been stored overnight in 4°C. We used anti-rabbit antibody for CD3 and anti-mouse for CD8, both with 1:200. Staining was performed with VECTASTAIN ABC-AP Staining KIT (AK-5000, Alkaline Phosphatase, Vector Laboratories Inc., USA) as described by manufacturer.

### IHC controls in HCC and PDAC

Haemotoxylin was used as a counterstaining for both frozen and paraffin sections. Positive and negative controls were performed as appropriate: tonsil tissue used as positive control. Quality control after immunohistochemistry was implemented according to Maxwell et al. [45]. The portion of cancer cells, the extent of necrosis, staining intensity, uniformity, specificity, absence of background staining, and counterstaining were quantified. Only optimal sections and stainings were permissible for this study [46].

### Picture capturing and analysis

The slides were visualized under the microscope (BX41, Olympus Corporation, Japan). Images of hot spots were captured with 200x enlargement using ZEN software (ZEN Version 2.0, Carl Zeiss Inc., Germany). 3 hot spots were evaluated in every slide. A hot spot was defined as the area with the highest density of infiltrating T-lymphocytes [9, 38], excluding lymph nodes. Included slides had at least one peri- or intratumoral hot spot. Biggest hot spots were selected by two blinded observers subjectively.

### Manual counting

We defined manual counting as the gold standard [14]. Using the ImageJ Software, the infiltrating immune cells were manually counted by the functions “Analyze” and “Cell Counter”.

### Algorithm

Reliability and accuracy of computed quantification was tested in order to develop a general algorithm usable for all tumor types analyzed: First, the reliability of identification of hot spots was investigated using two blinded observers (RCM, JH for HCC and PDAC; KD, FS for mCRC and OvCa). The absolute amounts of cells were compared with the intraclass correlation coefficient (ICC) to identify differences between two blinded observers.

Second, accuracy was tested. This experiment was divided in two parts. To examine whether quantification of 1 vs 3 hot spots yields accurate results the CD8^+^/CD3^+^ ratio as well as the absolute cell numbers were compared with the ICC respectively. Most authors described analysis of 3 hot spots [9, 15, 24]. Therefore, the most populated CD3^+^ hot spots of the slides were chosen, then the same hot spot was detected in the CD8^+^ slides and the ratio of CD8^+^/CD3^+^ cells was calculated.

Third, the following computerized counting methods: ZEN 2 software counting (ZC), ImageJ software (U. S. National Institutes of Health, USA) with subjective threshold (ISC) and ImageJ with color deconvolution (IAC) were compared to a manual counting (gold standard) using a linear regression analysis. Furthermore, duration to count one hot spot and costs were compared for every method.

### Automated ZEN 2 software counting (ZC)

The image analysis was configured by defining the measuring frame. Then, automatic segmentation by specification of the color spectrum was included. Finally, we defined the measurement features (scope, area, color spectrum, density, and watershed) and measured the stained cells. These steps were standardized for each antibody and each tumor type: the saved measurement features for CD3 and CD8 were used respectively.

### ImageJ with subjective threshold (ISC)

First, the original picture was changed to a 32-bit format and the subjective staining threshold defined using the standard ImageJ software. With the so called watershed function a separation of larger particles was performed. These particles were then automatically counted using the software function “analyze particles” for quantification.

### ImageJ with color deconvolution (IAC)

The color deconvolution application for ImageJ is freely available as an add on tool to the standard software [46]. The original picture of a hot spot was split in three color spectra. Furthermore, it was converted into a binary picture. Quantification of red particles is performed using watershed application of marked areas [47].

### Statistical analysis

For statistical analysis SPSS statistics software (SPSS Version 24.0, IBM Corporation, USA) was used. In the descriptive statistics the amount of infiltrating cells was calculated as median and range in quartiles. For comparison on the continuous scale the Mann-Whitney U Test was employed when appropriate (n_1_ + n_2_ > 30). A *p*-value of 0.05 was considered statistically significant. Reliability and accuracy were tested by linear regression and reliability analysis to present values of ICC and regression coefficient B.

## Abbreviations

mCRC: metastatic colorectal cancer
HCC: hepatocellular carcinoma
IAC: ImageJ with color deconvolution
ICC: intraclass correlation coefficient
IHC: immunohistochemistry
ISC: ImageJ software with subjective treshold
OvCa: ovarian cancer
PDAC: pancreatic ductal adenocarcinoma
QTiS: Quantification of the Tumor immune stroma
TMA: tissue microarray
TIL: Tumor-infiltrating lymphocyte
ZC: ZEN 2 software counting.
